# Tick-Borne Encephalitis Virus: A Comprehensive Review of Transmission, Pathogenesis, Epidemiology, Clinical Manifestations, Diagnosis, and Prevention

**DOI:** 10.3390/microorganisms11071634

**Published:** 2023-06-22

**Authors:** Emina Pustijanac, Moira Buršić, Jasminka Talapko, Ivana Škrlec, Tomislav Meštrović, Dubravka Lišnjić

**Affiliations:** 1 Faculty of Natural Sciences, Juraj Dobrila University of Pula, 52100 Pula, Croatia; 2 Faculty of Dental Medicine and Health, Josip Juraj Strossmayer University of Osijek, Crkvena 21, 31000 Osijek, Croatia; 3 University Centre Varaždin, University North, 42000 Varaždin, Croatia; 4 Institute for Health Metrics and Evaluation and the Department of Health Metrics Sciences, University of Washington, Seattle, WA 98195, USA; 5 Faculty of Medicine, Josip Juraj Strossmayer University of Osijek, Josipa Huttlera 4, 31000 Osijek, Croatia

**Keywords:** clinical manifestations, diagnosis of TBEV, epidemiology of TBEV, TBEV, tick-borne encephalitis virus, transmission and circulation in nature

## Abstract

Tick-borne encephalitis virus (TBEV), a member of the *Flaviviridae* family, can cause serious infection of the central nervous system in humans, resulting in potential neurological complications and fatal outcomes. TBEV is primarily transmitted to humans through infected tick bites, and the viral agent circulates between ticks and animals, such as deer and small mammals. The occurrence of the infection aligns with the seasonal activity of ticks. As no specific antiviral therapy exists for TBEV infection, treatment approaches primarily focus on symptomatic relief and support. Active immunization is highly effective, especially for individuals in endemic areas. The burden of TBEV infections is increasing, posing a growing health concern. Reported incidence rates rose from 0.4 to 0.9 cases per 100,000 people between 2015 and 2020. The Baltic and Central European countries have the highest incidence, but TBE is endemic across a wide geographic area. Various factors, including social and environmental aspects, improved medical awareness, and advanced diagnostics, have contributed to the observed increase. Diagnosing TBEV infection can be challenging due to the non-specific nature of the initial symptoms and potential co-infections. Accurate diagnosis is crucial for appropriate management, prevention of complications, and effective control measures. In this comprehensive review, we summarize the molecular structure of TBEV, its transmission and circulation in natural environments, the pathogenesis of TBEV infection, the epidemiology and global distribution of the virus, associated risk factors, clinical manifestations, and diagnostic approaches. By improving understanding of these aspects, we aim to enhance knowledge and promote strategies for timely and accurate diagnosis, appropriate management, and the implementation of effective control measures against TBEV infections.

## 1. Introduction

Tick-borne encephalitis virus (TBEV) is a member of the genus *Flavivirus* and belongs to the family *Flaviviridae* [1,2]. In humans, TBEV causes infection of the central nervous system, which can have serious consequences and lead to permanent neurological complications or even death [3,4]. The morbidity and mortality rates of tick-borne encephalitis (TBE) differ according to the three viral subtypes; namely, European (TBEV-Eu), Siberian (TBEV-Sib), and Far Eastern (TBEV-FE) [3,4,5,6]. In addition to the three main subtypes, two recently described subtypes have emerged. The first is the Baikalian subtype (TBEV-Bkl), consisting of 13 strains identified in east Siberia and northern Mongolia [7,8]. The second is the Himalayan subtype (TBEV-Him), which has been found in wild rodents in the Qinghai–Tibet Plateau region of China [9]. In the last three decades, the spread of TBE has become a substantial concern in Europe and Asia. Notably, there has been an expansion of TBE risk areas into regions that were previously unaffected alongside the emergence of new endemic areas [10,11]. The incidence of TBEV infections has been steadily increasing, posing a significant and growing health concern [2]. This comprehensive review thoroughly examines the molecular structure of TBEV, its transmission and circulation in natural environments, the pathogenesis of TBEV infection, the epidemiology and global distribution of the virus, associated risk factors, clinical manifestations, and diagnostic approaches.

## 2. An Insight into the Molecular Structure of Tick-Borne Encephalitis Virus

*Flaviviruses* undergo a maturation process during their production, giving rise to three distinct types of particles within infected cells: immature non-infectious particles, partially mature particles, and fully mature infectious particles [12,13,14]. Mature TBEV particles have a smooth, spherical morphology and are membrane-enveloped with a diameter of approximately 50 nm, similar to those of other Flaviviruses [12,15,16,17,18]. The icosahedral nucleocapsid, which measures about 30 nm in diameter, consists of several copies of a single viral capsid protein (C) and genomic RNA [13]. The nucleocapsid of TBEV is encased within a membrane. There is a distinction between mature and immature viral particles. Mature viral particles possess an envelope comprising envelope proteins (E) and membrane proteins (M). In contrast, intracellular immature viral particles contain the precursor M protein (prM) in place of the M protein. The prM protein is proteolytically cleaved before the virion is released from the host cell [1]. PrM acts as a chaperone that directs the proper folding of E protein. The envelope E protein creates rod-shaped dimers oriented parallel to the membrane, covering the surface of the viral particle. The mature TBEV particle envelope contains three E proteins and three M proteins in each icosahedral asymmetric unit [12]. The surface of the TBEV virion is adorned with small protrusions, which are created by glycans attached to the E protein subunits. A compact heterotetramer is formed by two E proteins and two M proteins. The envelopes of flaviviruses exhibit a herringbone pattern consisting of three of these heterotetramers [12]. The virus membrane does not have a spherical shape; instead, it closely conforms to the inner surface of the protein envelope. The membrane undergoes deformations due to the insertion of transmembrane helices from E proteins and M proteins [12]. The viral particles are composed of 6% ribonucleic acids (RNA), 66% proteins, 17% lipids derived from host cell membranes, and 9% carbohydrates [1,19].

### 2.1. Organization of the Genome

**Figure 1:**
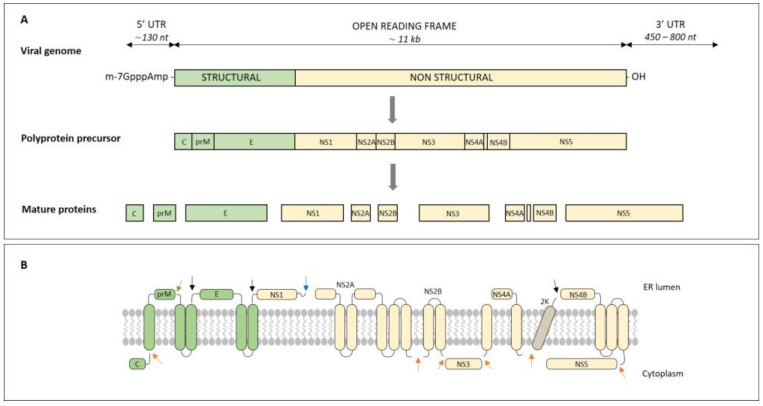
Genome organization of TBE virus (**A**) and the schematic representation of produced polyproteins with cleavage products (**B**). The structural proteins are represented in green, while the nonstructural proteins are in yellow. Cleavage sites for viral serine protease are indicated by black arrows, host signal peptidase cleavage sites by orange arrows, an unknown host protease cleavage site by a blue arrow, and a furin cleavage site by a green arrow.

The genome of the virus is a positive-sense single-stranded RNA, approximately 11,000 nucleotides long. Genomic RNA is infectious and is the only viral mRNA present in infected cells [1]. It consists of two short non-coding sequences at the 5′- and 3′- ends and one open-reading frame (ORF), which is approximately 11,000 nucleotides long. After translation and processing of the viral genome, 10 different proteins are produced, three structural (C, prM, and E) and seven nonstructural (NS1, NS2A, NS2B, NS3, NS4A, NS4B, and NS5) [20,21]. The 5′-short non-coding sequence is approximately 130 nucleotides long and contains a type-I cap (m-7GpppAmp) followed by two conserved AG dinucleotides. The length of the 3′-short non-coding sequence can vary from 450 to 800 nucleotides and it may contain an internal poly-A tail [1,22]. RNA noncoding sequences create secondary structures that participate as cis-regulatory elements in genome replication, translation, and viral particle assembly [22]. The virus genome has three different roles: (1) as messenger RNA (mRNA) for the translation of viral proteins, (2) as a template for RNA amplification, and (3) as genetic material stored in new viral particles (Figure 1) [23]. 

### 2.2. Viral Proteins

The virus genome is translated into a single polypeptide molecule (~3400 amino acids). The primary translation product is then co-translationally and post-translationally cleaved at specific sites by host and viral proteases. Thus, three structural and seven nonstructural proteins are produced, which participate in virus replication [22,24]. Host signal peptidases cleave the polyprotein at the C/prM, NS2A/NS2B, NS2B/NS3, NS3/NS4A, NS4A/2K, and NS4B/NS5 junctions. The serine protease of the virus cleaves the polyprotein at the junctions prM/E, E/NS1, and 2K/NS4B [25]. It is not known which enzyme is responsible for cleaving the contact between NS1 and 2A (Figure 1) [23].

#### 2.2.1. Structural Proteins

During polyprotein translation, structural proteins are anchored to the host’s endoplasmic reticulum (ER) with distinct signal sequences and transmembrane domains. The C protein contains a C-terminal hydrophobic signal sequence that translocates the prM protein into the ER lumen. The prM protein contains two transmembrane domains containing stop sequences and a signal sequence. As a result, the E protein is also translocated to the lumen of the ER. In the ER lumen, prM and E proteins form stable heterodimers [26,27]. 

Protein C (~120 amino acids, 11 kDa) is a small, strongly basic protein that forms a structural component of the nucleocapsid [23]. The basic building block of the capsid is a dimer of protein C. Each monomer consists of four different α-helices (α1 to α4) connected by short loops [28]. Helices α2 and α4 of one monomer are antiparallel to helices α2 and α4 of the other monomer. All four helices are part of the central hydrophobic domain responsible for interactions within the dimer. It is assumed that the α4-helix, which has a high content of positively charged amino acids, binds non-specifically to viral RNA. At each end of the dimer are two hydrophobic α2-helices that interact with the membrane surrounding the nucleocapsid and are within a groove flanked by the α1-helix [24,28]. The hydrophobic C terminus of the precursor protein anchors the C protein to the cytoplasmic side of the ER membrane and is an internal signal sequence that allows translocation of the prM protein into the ER lumen. The signal sequence is cleaved in the mature viral particle by the viral protease NS2B-NS3 [29]. Virus multiplication produces complete virus particles and a smaller number of sub-viral non-infectious particles. Deletions or alterations in the amino acid sequence of protein C can lead to the elevated presence of non-infectious sub-viral particles during the assembly of viral particles [29]. It has been observed that the prM and E proteins have the capability to independently assemble sub-viral particles that lack a nucleocapsid [1]. 

The prM protein (~165 residues, 26 kDa) is the glycosylated precursor of the structural M protein (~75 residues, 8 kDa). In immature viral particles, the prM protein forms heterodimeric complexes with the E protein and thereby protects it by preventing its fusion with the membrane of the trans-Golgi network, which would otherwise occur due to low pH. The prM protein acts as a chaperone for the proper assembly and folding of the E protein [26]. Prior to the release of immature virus particles from the host cell, the prM protein undergoes cleavage by the furin protease (Figure 1). This cleavage event separates the prM protein into two sections: the N-terminal section, known as the soluble ”pr”; and the C-terminal section comprising the structural M protein, which remains anchored to the cell membrane [30]. The M protein consists of a single peripheral membrane helix (h1), two transmembrane helices (h2 and h3), and an N-terminal loop region that engages in interactions with both E proteins. A heterotetramer is formed by two M proteins and two E proteins, wherein each M protein interacts with both E proteins [12]. The heterotetramer functions as the fundamental unit of the mature virion. The N-terminal loop of the M protein interacts with domain II of the E protein, which likely hinders the reorganization of E protein dimers into fusogenic trimers [17].

The E protein (~496 residues, 52 kDa) is the basic envelope protein that covers almost the entire outer surface of the mature viral particle, and it is, therefore, also the primary target of neutralizing antibodies. It is responsible for key functions related to virus entry into cells, such as receptor binding and membrane fusion, and induction of neutralizing antibodies [12,31]. It is thought that it can attach to several different host cell receptors in both vertebrates and different arthropods (ticks and mosquitoes) [24,32]. The E protein is an elongated molecule that, before fusion, has the form of a dimer, with the two monomers being oriented opposite to each other (the head of the first faces the tail of the second). On the surface of the virus, the E protein creates a unique so-called herringbone pattern. At low pH, the dimeric form of the E protein dissociates into monomers and then changes irreversibly to the more stable trimeric form [33]. The E protein monomer is comprised of four distinct domains. The N-terminal β-barrel domain (domain I) serves as a central structure within the protein [12,31]. The extended dimerization domain (domain II) of the mature virus contains the sole glycosylation site (Asn154). It is composed of two regions of β-strands connected by loops and two short helices. In addition to its function in dimerization, this domain also plays a crucial role in egress from mammalian cells and contributes to neurovirulence [34,35]. Domain II also encompasses the highly conserved fusion loop, which plays a crucial role in mediating the fusion of the viral and host membranes during the final stages of TBEV entry [12,31]. Domain III exhibits a characteristic immunoglobulin-like shape and has been suggested to play a crucial role in binding to host receptors [31]. Domain IV consists of a stem region comprising three peripheral membrane helices (h1–h3) and a transmembrane region consisting of two helices (h4 and h5) [12]. The connections between the domains are flexible, which is very important in the change from an immature to a mature viral particle, as well as in the process of fusion with the membrane [17,36]. The C terminus of the protein is anchored to the ER membrane. The transmembrane region of the E protein also plays an important role in the assembly of the viral particle and in the later stages of membrane fusion because it enables the necessary intra- and intermolecular contacts in E protein trimers [37]. The nucleotide sequence of protein E is both sufficiently long and, despite its diversity, adequately conserved for phylogenetic analyses [38]. Furthermore, the gene database contains a vast amount of information regarding numerous E protein records with origins spanning diverse geographic locations. Several mutations in domain III have been identified, which can significantly impact the neurovirulence and neuroinvasiveness of TBEV, Louping-ill virus, and Langat virus [39,40,41,42,43]. A single amino acid substitution in domains I, II, or III of the E protein transcript has the potential to result in the attenuation of neurovirulence or neuroinvasiveness [40,44,45,46,47]. 

#### 2.2.2. Nonstructural Proteins

The NS1 protein, consisting of 351 residues and weighing 46 kDa, is a glycoprotein known for its high conservation. During synthesis, it translocates to the ER. The host signal peptidase cleaves it from the E protein. Within infected cells, the NS1 protein serves as a cofactor in viral RNA replication. It has the ability to form homodimers, and when in the hexameric form (composed of three homodimers), it is released into the serum of patients. Hexameric NS1 proteins form a ring-like structure with a diameter of approximately 10 nm. They are internalized by hepatocytes and transported to late endosomes, where they accumulate and exert immunomodulatory activities [23,48,49,50]. The NS2A protein, comprising 229 residues and weighing 22 kDa, is a relatively small hydrophobic protein. It is believed to have a function in binding matrix RNA to the ER membrane within the replication complex [48]. The NS2B protein, consisting of 130 residues and weighing 14 kDa, is a small, membrane-bound protein. It forms a stable complex with the NS3 protein and acts as a cofactor for the serine protease activity of the NS3 protein. On the other hand, the NS3 protein, a large cytoplasmic protein of 621 residues weighing 70 kDa, associates with the membrane through its interaction with the NS2B protein. It plays a multifaceted role in viral replication, serving as a helicase, an RNA triphosphatase, and, when bound to the NS2B protein, a serine protease [51]. The NS2B-3 protease plays a crucial role in mediating most of the cleavages within the nonstructural region of the viral polyprotein, including the cleavage that releases the C protein from the transmembrane signal sequence. Additionally, during helicase activity, it utilizes the energy derived from the hydrolysis of nucleoside triphosphates (NTPs) to unwind the newly formed RNA from the matrix and unravel the secondary structures that mark the beginning of replication. Furthermore, it is involved in modifying the 5′ end of the genome through its RNA triphosphatase activity [51]. The NS4A protein, consisting of 149 residues and weighing 16 kDa, and the NS4B protein, composed of 252 residues and weighing 27 kDa, are small hydrophobic proteins. Their primary role is to anchor the polyprotein, which is synthesized during the translation of the virus genome, into intracellular membranes. This anchoring facilitates the proper functioning of the polymerase complex and ensures the accurate cleavage of the polyprotein [48,51]. The NS5 protein, comprising 902 residues and weighing 103 kDa, stands as the largest nonstructural protein found in flaviviruses. It holds the distinction of being the most conserved and stable component of the viral genome. Playing a pivotal role, it serves as a central hub for viral RNA replication. The NS5 protein encompasses viral RNA-dependent RNA polymerase activity and exhibits methyltransferase activity, crucial for stabilizing and translating the RNA molecule. Moreover, it actively participates in suppressing the immune response, making it a significant virulence factor [52,53].

### 2.3. Multiplication of the Virus

**Figure 2:**
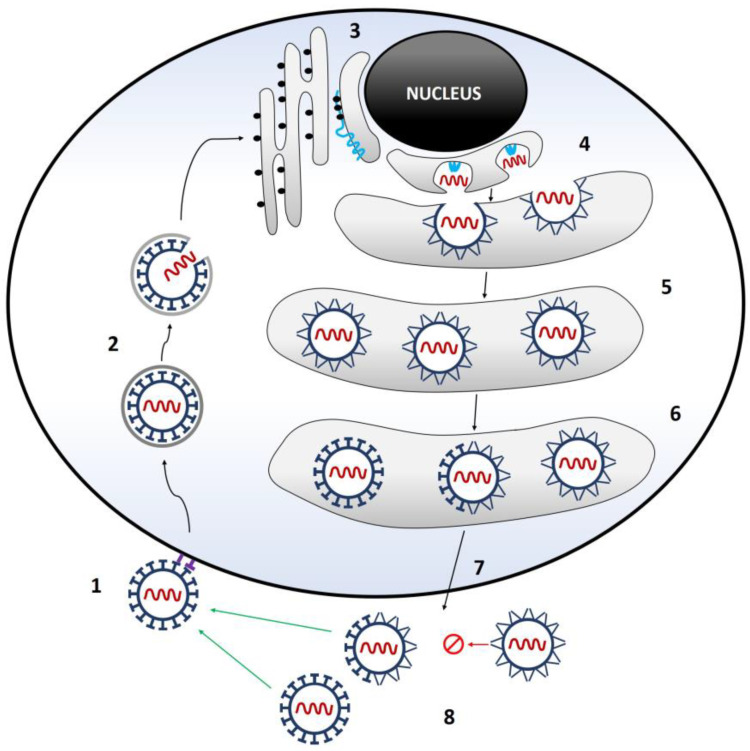
TBEV life cycle overview. The virus attaches to a receptor on the surface of a cell and enters the cell through endocytosis (1). Once inside the cell, the acidic environment of the late endosome triggers the fusion of the viral and endosomal membranes, leading to the uncoating of the virus (2). The cell ribosomes of the rough endoplasmic reticulum (ER) synthesize viral proteins (3). The virus replicates its genetic material within invaginations induced by the virus in the ER. The newly synthesized genomes are subsequently captured by the C protein on the cytoplasmic side of the ER (4). The nucleocapsid complex, comprising the viral genetic material, obtains structural E and M proteins, along with a lipid envelope, by budding into the ER lumen through the membrane (5). The immature viral particles are transported through the Golgi network where they undergo maturation in the acidic trans-Golgi environment (6). The mature viral particles, partially mature and immature particles, are released from the infected cell (7). While the mature and partially mature particles can initiate a new infection cycle, the immature particles are non-infectious because they are unable to fuse with other cells (8).

The genome of viruses with positive-polar single-stranded RNA simultaneously enables the transfer of information embedded in RNA from one generation of viruses to another and the formation of proteins in the process of translation. The initial stage of TBEV replication involves the attachment of the virus to the host cell surface. This attachment is facilitated through receptor-mediated binding of virus particles to cells [54,55,56]. The association occurs between the viral envelope E protein and the cellular glycosaminoglycan heparan sulfate, which is abundantly represented on the membranes of various vertebrate and tick cells [32,57,58]. The primary mode of entry for the virus into host cells is through receptor-mediated endocytosis, although entry through micropinocytosis is also possible [25,59,60,61]. Initially, the virus is localized within the prelysosomal endocytic vesicle of the host cell. The acidic pH environment within the endosomes triggers conformational changes in the envelope E protein, causing its redistribution. This event ultimately results in the fusion of the viral membrane with the membrane of the endocytic vesicle [62,63]. Upon entering the host cytoplasm, the nucleocapsid releases the genomic RNA. The process of viral protein synthesis is initiated through RNA translation, followed by replication of the viral genome. Replication of the viral genome involves two steps: amplification of the genomic RNA into a negative-polar RNA copy, which serves as a template, and subsequent transcription of the positive-polar RNA by the RNA-dependent RNA polymerase (RNA replicase). The positive-polar RNA is complementary to the genomic RNA. The genome of TBEV is initially translated at the ER as a single polyprotein. This polyprotein undergoes subsequent cleavage by both viral and host enzymes, leading to the generation of both structural and nonstructural proteins [13]. After translation, the prM and E proteins undergo transport into the ER lumen. The nucleocapsid, composed of several copies of the C protein and one copy of the genomic RNA, forms on the cytoplasmic side of the ER membrane. Subsequently, the nucleocapsid buds from the ER, acquiring a lipid envelope in the process (Figure 2) [64].

Non-infectious, immature virus particles are produced, their surfaces are covered with trimers of prM and E proteins, and they are easily transmitted through the host’s secretory pathway. Intact prM peptides cover the fusion loops of the E proteins and thus prevent fusion of the virus with intracellular membranes [65]. In addition, sub-viral particles that lack the nucleocapsid are also produced in the ER. Even after maturation, sub-viral particles remain non-infectious [29]. Viral maturation occurs when a host protease cleaves prM in cisternae of the trans-Golgi network [30,65]. The low pH induces a redistribution of the E protein that generates fusion-competent homodimers in a herringbone-like arrangement and the pr peptides docked onto the fusion loops, a configuration similar to that of the mature virion [65]. Following the cleavage of prM, the dissociation of pr peptides is pH-dependent, indicating that pr is retained on the virion within the acidic environment of the trans-Golgi network. This retention serves to prevent premature membrane fusion [66]. Upon release of the virions from infected cells into the extracellular space with a neutral pH, the pr peptides dissociate from the viral particles. This dissociation leads to the maturation of the virions, making them fusion-competent [65,67,68,69]. Mature, infectious viruses that express proteins M and E on their surface are released from the cell by exocytosis (Figure 2) [20,23,24,69,70]. 

## 3. TBE Virus Transmission and Circulation in Nature

TBE is a viral disease caused by the TBEV and is mainly transmitted to humans through the bite of infected ticks. In nature, TBEV is primarily transmitted between ticks, which serve as vectors, and animals such as deer and small mammals (for example, rodents and insectivores) that serve as reservoir hosts [71,72]. Small mammals are considered competent reservoir hosts, meaning that they can efficiently harbor and transmit the virus. In contrast, larger mammals typically develop only a brief period of viremia with low viral concentrations or no detectable viremia. As a result, these accidental hosts are not capable of effectively transmitting and spreading the virus [73].

Ticks acquire the virus by feeding on infected individuals and, when an infected tick bites a mammalian host, the virus can enter the host’s bloodstream and cause infection. The virus can then be transmitted to other animals or humans during subsequent tick feedings [74]. This is because the virus can be present in the tick’s saliva, which can enter the host’s bloodstream during the feeding process. In fact, transmission of TBEV from an infected tick to a host can occur within minutes to a few hours of tick attachment [75]. The longer an infected tick remains attached to a host, the greater the likelihood that tick-borne pathogens will be transmitted. Nevertheless, if an infected tick detaches from its initial host before it has fully fed and then attaches to a new host to complete its feeding, the transmission time may be reduced [74]. Ticks can also harbor the virus for extended periods of time. Once infected, they retain the infection for their entire life and can transmit the virus to other ticks or to vertebrate hosts [76]. This poses a concern because, even if a tick becomes infected early in the season, it can still transmit the virus to a host later on, particularly given that certain ticks have a lifespan of several years. For instance, the *Ixodes ricinus* tick has a potential lifespan of up to 8 years [77].

The geographic distribution of TBEV is largely determined by the distribution of its tick vectors. In Europe, the main vectors for TBEV-Eu are *Ixodes ricinus* ticks, while for TBEV-Sib and TBEV-FE, the main vector is *Ixodes persulcatus* [78]. The virus is also found in China, Mongolia, and parts of Russia and Scandinavia [79]. *Haemaphysalis concinna* appears to be a significant vector for the transmission of TBEV in Asia [80,81]. Additionally, studies have identified at least 22 other tick species that are capable of carrying and transmitting the virus that belong to the genera *Dermacentor*, *Hyalomma*, and *Rhipicephalus*, along with the aforementioned *Ixodes* and *Haemaphysalis* genera [72,82]. These ticks, which are external parasites, inhabit vegetation and actively seek hosts. As they progress through their life cycle (larva–nymph–adult), they move between different host groups, predominantly comprising diverse species of birds and mammals. Through this mechanism, they can transmit TBEV and promote its circulation in nature [83]. *I. ricinus*, which is found throughout Europe, has expanded its distribution to encompass extreme altitudes and latitudes [84]. It infests various vertebrate species throughout its life cycle. This species is found in diverse habitats, including deciduous and mixed forests, shrublands, urban parks, and gardens, with increasing population densities in urban and suburban areas [71]. On the other hand, the range of *I. persulcatus* covers Eastern Europe, Asia, China, and Japan [85]. Unlike *I. ricinus* nymphs, *I. persulcatus* nymphs infrequently feed on larger hosts, such as humans. These two species have contrasting temperature-related activity, as *I. persulcatus* is active in lower temperature ranges. *I. persulcatus* is typically found in regions with lower levels of precipitation, humidity, and temperature compared to the northern distribution limits of *I. ricinus* [86]. Host-seeking ticks of the *Haemaphysalis* genus seldom inhabit the same areas simultaneously, primarily due to differences in their habitat preferences, host choices, and seasonal patterns. *H. concinna* has a broad distribution in temperate Eurasia and typically occupies light and humid deciduous forests, as well as mixed hornbeam-oak forests [83]. Given its rapid reproduction rate, quick development cycle, and extensive host range, *D. reticulatus* has gained recognition for its significant involvement in transmitting TBEV. Its habitat spans a vast area from Portugal in the west to central Asia in the east. In Europe, its geographic distribution largely coincides with that of *I. ricinus*. There is a growing body of evidence reporting the detection of TBEV in *D. reticulatus*, with Poland and Germany being notable hotspots for such findings [87].

**Figure 3:**
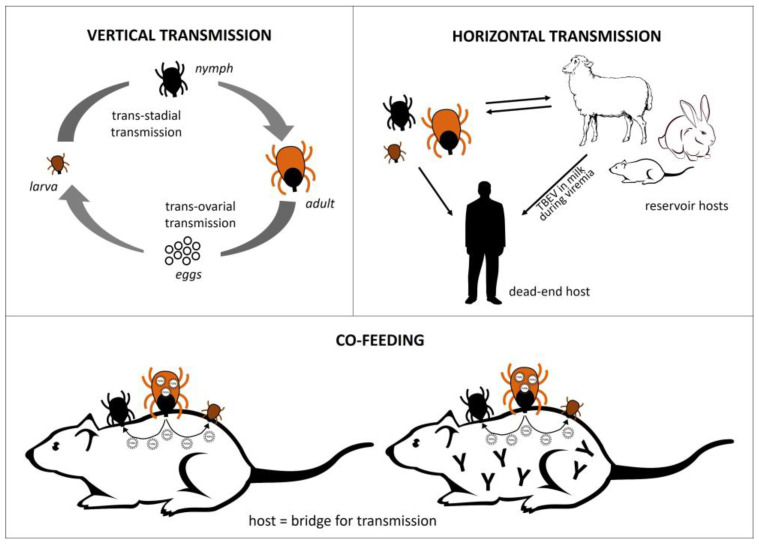
TBEV transmission pathways. In vertical transmission, infected ticks pass the virus to their offspring through eggs and across different life stages. Horizontal transmission occurs when uninfected ticks feed on infected vertebrate hosts, but humans are considered dead-end hosts as they do not develop the high levels of viremia necessary for the virus to be transmitted to ticks. In co-feeding, transmission occurs when infected and naïve ticks feed in close proximity on an animal host, which serves as a bridge for virus transmission. This method allows for the fast transmission of the virus among ticks, even if the host has antibodies against TBEV.

TBEV is maintained in tick populations through vertical transmission, which involves both trans-ovarial and trans-stadial routes. Trans-ovarial transmission occurs when an infected tick passes the virus to its offspring through the eggs, while trans-stadial transmission allows the virus to persist in the infected tick through all life stages; namely, the egg, larval, nymphal, and adult stages [88]. On the other hand, horizontal transmission is an important mechanism for the longevity and more effective spread of TBEV within tick populations and can also facilitate transmission to other hosts, including humans. Horizontal transmission happens when an uninfected tick feeds on an infected vertebrate host [76]. To enable transmission, a high level of viremia in the host is necessary, which occurs only in mammals such as sheep, goats, horses, dogs, and rodents. Humans do not develop high viremia, which would allow the virus to be transmitted to ticks, and that is why they are considered as the dead-end hosts of TBEV (Figure 3) [88].

Ticks are capable of transmitting TBEV through a process called co-feeding, which does not require a viremic host. This process occurs when naïve ticks feed in close proximity to an infected tick, and the animal host serves as a bridge for transmission [89]. Many tick species exhibit a noticeable spatial clustering on specific parts of the host body. In fact, approximately 90% of the immature stages (larvae and nymphs) of several tick species that feed on rodents attach to the ears, around the eyes, or on the snout, resulting in at least 45% of feeding ticks being within approximately 1 cm of each other [90]. This transmission can still happen even if the host has antibodies against TBEV, so co-feeding allows the virus to spread rapidly through a population of ticks, even when the prevalence of systemic infections in the host population is low (Figure 2) [72].

Another factor that stimulates TBEV transmission is climate change. As temperatures rises due to climate change, it is anticipated that the potential for transmission will also increase. This will be aided by an expected lengthening of the tick questing season, as well as increases in the number of susceptible ticks (in their larval and nymphal stages) and the number of infected nymphal ticks that co-feed on the same hosts. These factors will lead to a compounded increase in infections through non-systemic transmission [91].

### 3.1. Circulation of TBE Virus between Small Mammals and Birds

The virus can be transmitted between rodents through either vertical or horizontal transmission. Vertical transmission can occur during the prenatal phase through transplacental transmission or during the postnatal phase when newborns consume infected milk from their mothers. In contrast, horizontal transmission occurs in the wild during mating [92].

In rodents, viremia is short-lived, but it is high enough for ticks to become infected through feeding. After infection with TBEV, the host develops specific antibodies against the virus. Therefore, older, seropositive (immune) animals are not a suitable reservoir for the virus. However, in these individuals, the virus can still be transmitted between infected and uninfected ticks through co-feeding [93].

Ticks on birds are mainly acquired during feeding on the ground, and birds can transport these ticks to new areas that are inaccessible to ground-dwelling animals due to their ability to fly over barriers. If the transported tick encounters suitable environmental conditions, it can transmit the pathogen it carries. Birds are thought to play a primary role in spreading the virus to new endemic areas, similar to other tick-borne pathogens [94]. Long-distance dispersion of TBEV by birds is unlikely due to the short feeding period of Ixodes ticks on their avian hosts, which limits the distance covered while attached to the bird [72].

### 3.2. Circulation of TBE Virus between Humans

Ticks at all developmental stages can transmit the virus to humans. Unlike rodents, humans do not develop high enough viremia to become infectious for ticks [95]. Ticks usually do not feed on humans in large numbers or closely enough to each other to allow transmission through co-feeding [96].

The occurrence of TBEV infections in humans is largely influenced by the local prevalence of infected ticks, as well as the frequency of interaction between humans and these ticks. In areas where TBEV is endemic, the incidence of human infections can significantly fluctuate from year to year owing to changes in tick populations and variations in the virus prevalence within the ticks [97]. 

A person can also become infected by consuming unpasteurized milk and dairy products because domestic animals (cows, goats, sheep) excrete TBEV in their milk during viremia. This can happen during a period ranging from 3 to 14 days, with excretion beginning as early as the second or third day following infection [98].

Isolated instances of TBEV infection have been reported in patients receiving solid organ transplants [99] and blood transfusions [100], as well as in laboratory workers infected through the respiratory tract or oropharyngeal epithelial cells [101]. Inhalation of an infectious aerosol has also been mentioned as a possible route of infection, as well as from a puncture with a contaminated needle [102]. The interhuman transmission of TBEV through breast milk has neither been confirmed nor excluded. Nonetheless, there is a reported case where an unvaccinated mother probably transmitted TBEV to her infant through breastfeeding [103].

Preventing TBEV infections in humans primarily involves reducing the risk of tick bites through measures such as wearing protective clothing, using insect repellent, and checking for ticks after spending time outdoors. Vaccines against TBEV are also available and are recommended for individuals living in or traveling to areas where the virus is endemic [79].

## 4. Pathogenesis of Tick-Borne Encephalitis Virus Infection

On its way from the vector—in this case, the tick—to causing disease in humans, the TBEV travels a path with numerous obstacles [104]. Namely, when a tick infected with TBEV, in search of food, bites a human, it essentially pierces the skin, which represents the first protective barrier [105]. During feeding, the tick injects viral particles with its saliva that replicate at the point of entry into the body subcutaneously in skin neutrophils and Langerhans cells [106].

Macrophages (migrating monocytes) transport viral particles to regional lymph nodes, where they replicate, resulting in viremia. The virus continues to spread via the hematogenous route and reaches the reticuloendothelial system (the spleen, liver, and bone marrow), where, as a result of further multiplication, the viremia is maintained for several days [3]. TBEV causes changes in host cell membranes that create isolated viral factories, which are believed to shield viral RNAs from host defense mechanisms [107]. Furthermore, infection with TBEV activates innate immune signaling by engaging with the pattern recognition receptor RIG-I/MDA5, leading to the translocation of the interferon regulatory factor 3 (IRF-3) to the nucleus [107].

During viremia, tick-borne encephalitis virus passes the blood–brain barrier (BBB) and thereby enters the central nervous system (CNS) [108]. Neurons are the primary targets of TBEV infection in the CNS, but it is important to note that the exact mode of entry into the CNS and the cell receptors are still not fully understood [109,110]. Various receptors have been demonstrated in vertebrate cells in vitro, such as the laminin receptor in human embryonic cells and glycosaminoglycans (heparan sulfate) [25]. The exact mechanism by which TBEV penetrates the blood–brain barrier is not known, but four possible pathways have been hypothesized [111] (Table 1).

Studies performed on neurons and astrocytes differentiated from human neural progenitor cells (hNPCs) have shown that this cell type is permissive of TBEV infection [113].

Viral infection induces the death of neurons and astrocytes and the production of type-I interferons (type-I IFNs), which are involved in the control of viral replication and cell protection [114]. On its way to causing infection, TBEV encounters another obstacle, which involves the immune response to the presence of the virus [115]. Neurotropic viruses such as TBEV can trigger a wide range of immune responses, which can be associated with severe clinical outcomes [116]. The clinical outcome of TBEV infection depends on the immune response at the site of virus entry, the extent of peripheral infection and associated inflammation, and the humoral response [117,118].

Dendritic cells (DCs) are the first cells of the immune system to encounter TBEV after infection. In this first phase, DCs play an extremely important role in initiating the innate immune response, which includes the production of IFN-α and the presentation of the antigen (Ag) [119]. It is important to note that DCs, at the point of TBEV entry, can be influenced by some of the proteins found in tick saliva, which have immunomodulatory effects [120]. Namely, as a result of the recorded disturbance of the Th1-type cytokines (important for the removal of TBEV from the CNS), polarization, and the induction of the Th2-type cytokines’ response to and inhibition of DC maturation, we are led to conclude that the effect of the tick saliva on DCs is complex and may represent an extremely important mechanism of immune evasion mediated by ticks [121]. After the TBEV is taken up by the DCs, DC maturation occurs, resulting in the production of pro-inflammatory cytokines, chemokines, and type-I IFNs [115].

Macrophages represent another target of TBEV [122]. They function as antigen-presenting cells and have the main protective function. T and B cells located in the secondary lymphatic organs reveal the decomposed TBEV and are responsible for clearing TBEV from the circulation [116]. Recent studies have found a temporal disconnect between the highest levels of the virus in the bloodstream (i.e., peak viremia), the peak production of antibodies, and the onset of clinical symptoms in the disease [123]. More specifically, low IgG antibody response in the serum against TBEV during the initial stages of neurological symptoms usually translates to more challenging clinical progression and an adverse long-term outcome [123].

Likewise, natural killer (NK) cells participate in the recognition and removal of cells infected with TBEV, but their role has not been fully elucidated [118]. Despite this, it is known that NK cells, similarly to T cells, can participate in immunopathological reactions; therefore, they can directly kill infected cells and they can act indirectly with the help of cytokines or chemokines, but they can also recruit inflammatory cells in the tissue [118]. In addition, they can have a protective effect [117]. It is assumed that both mechanisms (immunological and non-immunological) can contribute to the invasion of the CNS by TBEV by enabling the passage of TBEV across the BBB [124]. This process can be facilitated by the cytokines TNF-α and IL-6 because they can cause disruption of the BBB due to their modulatory effect on the permeability of endothelial cells [111].

One of the ways for TBEV to cross the BBB is via a special “Trojan horse” mechanism, which represents the migration of infected immune cells, such as DCs, macrophages, neutrophils, monocytes, and T cells with TBEV, into the CNS, where they lead to the infection of neurons and of other cells in the spinal cord and brain [112]. Likewise, infection and replication of TBEV in the epithelial cells of the choroid plexus or endothelial cells and virus budding on the parenchyma side is a possible mode of entry into the CNS [125]. A putative alternative pathway that can lead to CNS infection with TBEV, which does not involve the BBB, is through the invasion of the olfactory epithelium by TBEV, which is followed by infection of the olfactory neurons and, ultimately, entry into the CNS [111].

The intestinal route of infection with TBEV is associated with the consumption of thermally untreated milk and milk products from infected animals (sheep, goats, cows) [126]. In normal gastric juice, TBEV is stable for up to two hours [101]. DCs are the first cells of the immune system that come into contact with TBEV after its entry into the digestive system; that is, TBEV is transferred to DCs from intestinal epithelial cells that are infected with TBEV [117]. It should certainly be noted that this route of infection results in an increased immune response, resulting in a milder form of the disease “biphasic milk fever” [101].

## 5. Epidemiology of TBE Virus Infection: Global Distribution and Risk Factors

TBE is endemic in a wide area from Central Europe and the Scandinavian Peninsula to Japan [3]. However, the highest incidence has been documented in the Baltic and Central European countries [127]. In addition, the indigenous occurrence of the infection in humans and animals, the isolation of the TBE virus from ticks, and the specific antibodies to the TBE virus detected in wild and domestic animals suggest an endemic TBE area [128]. The distribution areas of TBE range from the Bordeaux region of France in the west through Italy in the south and the Scandinavian Peninsula in the north to Siberia, China, and Japan in the east [129]. In the last two decades, an increase in TBE incidence has been observed in endemic areas but also the occurrence of sporadic cases outside endemic areas [130]. New endemic areas have been discovered in the Netherlands, England [11,131], South Korea [132], Mongolia, Denmark, high-altitude Kazakhstan, Kyrgyzstan, parts of Armenia, Azerbaijan, and Uzbekistan [128]. 

The incidence of TBE increased to 0.9 cases per 100,000 people in 2020 [130], an increase from rates of 0.7 in 2019 and 0.4 in 2015 [127]. The increase in the reported incidence rates is likely the result of a combination of social and environmental factors, as well as increased medical awareness and advanced diagnostics [133]. Three subtypes of the TBE virus have been well-described, the European, Siberian, and Far Eastern subtypes, and two additional subtypes have been proposed: Himalayan and Baikal subtypes [130]. The European subtype of the TBE virus occurs in Europe but has also been detected in the western Urals and Siberia. In contrast, the Siberian subtype of the TBE virus occurs in the Baltic states, northern Finland, and Siberia. The Far Eastern subtype of the TBE virus is endemic in Far Eastern Asia and Japan and has also been detected in central and eastern Siberia [3]. The European countries with the highest incidence of the disease in 2016–2020 were Lithuania (24.3 cases per 100,000 inhabitants per year), Slovenia (8.9), the Czech Republic (7.9), and Latvia (7.8) [127]. 

TBE virus infection is characterized by seasonal occurrence, which depends on the activity of the vector. In Central Europe, *I. ricinus* tick activity is highest in April and May and then again in September and October, while *I. persulcatus* is most active in the Urals, Siberia, and the Far East from late April to early June [3,130,134]. Reported infection rates for *I. ricinus* range from 0.1% to 5%, while in endemic areas of Siberia, approximately 40% of *I. persulcatus* ticks carry the virus [3,135]. In certain European endemic areas, such as Northern Europe and some Central European countries (for example, Germany, the Czech Republic, and Poland), the annual maximum of TBE cases occurs only in the summer months from July to August. About 95% of cases are diagnosed between May and November, with a peak between June and August [130]. People from rural areas, forest workers, hunters, soldiers, and others who frequently spend time in nature, such as picnickers, are more likely to become infected [2,136]. Therefore, it is not surprising that the number of reported cases is higher in men (male-to-female ratio: 1.5:1) and in the age group of 45 to 64 years [127].

A known route of infection is the consumption of unpasteurized milk or milk products from cattle exposed to ticks in endemic areas [137]. Most cases have been recorded during the summer months and have been associated with the consumption of goat milk products [138]. TBE outbreaks via food have been reported mainly in highly endemic countries, such as the Czech Republic, Poland, Hungary, and Slovakia [130]. The largest known foodborne TBE outbreak occurred in Czechoslovakia in 1954, when more than 600 people became infected with TBE after consuming TBEV-contaminated goat milk [138].

Several factors have influenced the increase in the occurrence of TBE in endemic areas and its spread outside endemic areas [139,140]. First, climate change (e.g., global warming) is leading to increases in TBEV reservoir and vector populations [141]. Temporal and spatial changes in temperature, precipitation, and humidity have significant impacts on tick biology and ecology [134,142,143]. Mild winters and an earlier onset of spring can increase host populations and accelerate tick development [78,144]. Due to climate change, the incidence of TBE and its spread to higher altitudes are increasing [53,137]. Second, TBE is travel-dependent and depends on the travel season and consumption of unpasteurized milk and milk products in endemic areas. Cases have been reported where the disease has been introduced to countries outside known endemic areas [128]. Approximately 1 to 2% of TBE cases are considered travel-related and pose an increased health risk to travelers [143]. Third, population migration to the suburbs and changes in lifestyle habits, as well as more frequent outdoor visits and recreational activities, lead to an increased likelihood of people coming into contact with infected ticks [144,145]. Finally, the number of infectious diseases in Europe decreased during the COVID-19 pandemic, with the exception of TBE, cases of which increased in 2020 compared to the previous three years [146,147]. Increased human outdoor activity during the pandemic and increased numbers of ticks may have contributed to this trend [144]. Some countries experienced a decrease in TBE cases during the COVID-19 pandemic (Sweden and Poland). The reason could be the more limited availability of diagnostic tests for TBE and the overload of the healthcare system during the pandemic [144,148].

**Figure 4:**
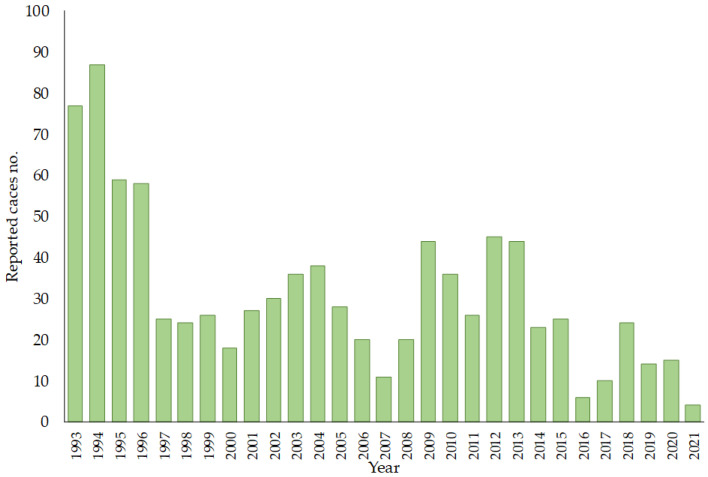
Numbers of reported TBE cases in Croatia from 1993 to 2021.

TBE was first described in Croatia in 1953, and Croatia is one of the countries with a low incidence of TBE [149,150]. TBEV has also been detected in ticks collected from natural hotspots in northwestern and eastern Croatia [151]. All TBE viruses detected in Croatia belong to the European subtype of the TBE virus and are genetically very similar, confirming that the disease is endemic to mainland Croatia [152]. From 1993 to 2021, a total of 900 TBE cases were reported in Croatia ranging from 6 to 87 per year, corresponding to an incidence of 0.10 to 1.90 cases per 100,000 population [153] (Figure 4). The highest TBE incidence was recorded in the northwestern counties of Croatia and in the age group of 50–59 years. Most infections (73%) were recorded from May to July [154]. There are also reports of foodborne TBE in Croatia. In 2015, 9 of 26 TBE cases were foodborne [149], and in 2019, 5 of 13 TBE cases were foodborne [155]. All involved consumption of unpasteurized goat milk or cheese from local farms but in two different parts of Croatia: the northwest and the Gorski Kotar region.

## 6. Clinical Manifestations, Treatment, and Prevention of Tick-Borne Encephalitis Virus Infection

TBEV infection may present clinically as a mild, abortive, non-specific febrile illness; a moderately severe illness with a biphasic course affecting the CNS; or a very severe disease with complications and permanent neurological sequelae but also a possibly fatal outcome [3,136,156,157].

Over 70% of infections attributed to the European TBEV subtype are asymptomatic. In the remaining cases, after an incubation period of 7–14 days (with a range of 1–28 days), the classical biphasic course of the disease develops. This also occurs after the consumption of unpasteurized domestic milk and milk products obtained from infected sheep and goats, with symptoms appearing within 3–4 days [3,136,149,156,157].

TBEV infection progresses through distinct phases, each characterized by specific clinical manifestations. The initial phase, known as the viremic prodromal phase, typically lasts for several days. During this phase, non-specific symptoms resembling a flu-like syndrome are observed, including fever, mild headache, general malaise, myalgia, arthralgia, and nausea. Following the prodromal phase, an asymptomatic interval follows, which can last anywhere from 1 to 21 days. In approximately 20–30% of TBEV-infected patients, a second phase occurs marked by the recurrence of fever and signs of CNS involvement, which vary based on the affected area and can include meningitis, meningoencephalitis, myelitis, and radiculitis. It is crucial to emphasize that TBEV infection is considered one of the most severe neuroinfections. The neurological complications that arise during the second phase can have long-lasting effects, leading to permanent neurological sequelae in some cases [3,22,136,156,157,158,159,160,161]. 

Meningitis is a significant manifestation observed in approximately 50% of patients infected with TBEV, with children being more commonly affected. It is characterized by fever, headache, vomiting, neck stiffness, and photophobia [3,136,156,157,159,162].

Approximately 40% of patients develop encephalitis, which can manifest as altered consciousness (such as somnolence, sopor, and, less commonly, coma), restlessness, agitation, tremor, ataxia, fasciculations of the tongue, dysarthria, paresthesia, focal or generalized seizures, muscle pain in the affected areas, paralysis (usually affecting the shoulder region and arms due to the virus’s preference for the anterior horns of the cervical part of the spinal cord), and potential respiratory muscle paralysis [2,3,136,151,156,157,159,160,162,163]. 

Myelitis occurs in approximately 5–10% of patients infected with TBEV. It commonly presents in conjunction with encephalitis and, rarely, as a separate clinical entity [2,3,159,162].

When TBEV affects the brainstem and medulla oblongata, it poses a high risk of mortality due to severe complications. Although less common, TBEV can also lead to polyradiculitis and cranial neuritis. While less frequent than encephalitis and myelitis, these conditions can still cause significant morbidity. Rare cases of TBEV-associated myocarditis, pancreatitis, and hepatitis have been reported, indicating the systemic impact of the infection beyond the central nervous system [136,164].

While most patients infected with TBEV experience complete recovery, up to 20% of cases can develop post-encephalitic syndrome. This syndrome is characterized by a range of long-term neurological and psychological sequelae that significantly impact the affected individuals’ quality of life. Some common manifestations of post-encephalitic syndrome include a persistent headache, weakening or loss of hearing, tinnitus, ataxia, uncoordinated movements, cognitive function impairment, depression, anxiety, and contractures [3,136,156,157,162,165].

In a small subset of patients comprising approximately 2% of cases, a particularly severe clinical course of the disease can lead to a fatal outcome. This subset is characterized by factors such as older age, the presence of comorbidities, and a monophasic course of the illness [3,159,165,166].

### 6.1. Treatment

As there is currently no specific antiviral therapy available for TBEV infection, the treatment primarily focuses on symptomatic and supportive measures. Patients with a severe and complicated course of the disease receive care in intensive care units, where a comprehensive approach is taken to manage their condition. Treatment in these cases typically involves supplementation of fluids, correction of electrolyte disturbances, antipyretics, anticonvulsants, and antiedematous treatment (mannitol, corticosteroids, assisted ventilation in cases of respiratory failure, prevention of decubitus, contracture, etc.) [3,156,157].

Studies have indicated that the use of interferon alpha and oral ribavirin has not demonstrated effectiveness in influencing the course of TBEV infection or promoting recovery [156,157]. Tetracyclines are occasionally employed for immunomodulation, primarily to inhibit the production of pro-inflammatory cytokines [156]. 

#### Immunotherapy

Due to concerns about potential risks and harm associated with immunotherapy, including the possibility of antibody-dependent enhancement of infection and the potential for long-lasting disabilities, the routine use of specific TBE immunoglobulins for prophylaxis and treatment of TBE infections is generally not recommended. The clinical and therapeutic approach remains highly complex, particularly when dealing with immunocompromised patients [79,167,168].

Over the past two decades, there has been growing utilization of rituximab (RTX) and other B-cell-depleting monoclonal antibodies (such as ocrelizumab, veltuzumab, and ublituximab) in the treatment of oncological, rheumatological, and neurological conditions [167,169,170]. The immunomodulatory effect of RTX is attributed to its extremely rapid action, leading to B-cell depletion within 2–3 days of administration. This effect is sustained, lasting up to 12 months [169,170,171]. The significant level of immunosuppression observed in these patients indicates that they may be highly vulnerable to severe infectious diseases. Examples include viral infections, such as enteroviruses, reactivation of herpes viruses, hepatitis B virus, and JC polyomavirus, as well as the devastating arboviral neuroinvasive disease, which has a high fatality rate. It is concerning to note that, among patients who undergo RTX treatment and are diagnosed with arboviral neuroinvasive disease, four out of five do not survive. Even for those who do survive, long-term disabilities are often present [167,170,172]. Experimental data strongly support the notion that antibodies and B-cells play a critical role in the prevention and containment of the early neurological dissemination of West Nile virus [173]. The identification of a recent cluster of fatal TBEV infections in patients who had undergone solid organ transplantation highlights the possibility that various types of immunosuppression could be a risk factor for more severe TBEV disease [167,174].

The diagnosis of arboviral infection can be challenging and delayed in patients undergoing RTX treatment, especially when the disease presents with atypical, prolonged, or subclinical symptoms. In these patients, the antibody response is often impaired or completely abolished, making it necessary to rely on molecular testing to detect viral RNA in serum, cerebrospinal fluid (CSF), and/or tissue specimens for an accurate diagnosis [167,170,171].

One potential immunotherapy approach for arboviral neuroinvasive diseases involves the administration of high doses of intravenous immunoglobulins (IVIGs). However, the efficacy of IVIG therapy remains uncertain due to heterogeneous data collected from case reports, case series, and observational studies. While the application of IVIG appears to be generally safe, there is no clear evidence of its superiority over supportive treatment [79,167,168,175]. In the case of West Nile virus (WNV) infection, high-dose IVIG has been used in patients treated with RTX, but they have not shown a definite clinical benefit [2,6]. Similarly, in a double-blind, placebo-controlled trial involving patients with Japanese encephalitis, no significant difference in outcomes was observed between the IVIG and placebo treatment groups [176].

Case reports have shown a strong correlation between fatal outcomes and pre-existing immunosuppression in arboviral neuroinvasive diseases [171,175]. The absence of intrathecal neutralizing antibodies likely leads to prolonged viral spread, as supported by the detection of TBEV RNA in the CSF of these patients. However, in cases where there is substantial viral replication in the CNS and a concurrent lack of endogenous TBEV antibodies, the administration of passively transferred neutralizing antibodies may help alleviate CNS disease and improve patient outcomes [167,173].

The increasing number of case reports documenting successful treatment of arboviral encephalitis with high doses of IVIG is encouraging [79,168,175,177,178]. IVIG has been utilized in the treatment of severe TBE. While some symptoms improved after treatment, neurological symptoms did not show the same level of improvement. There is a trend suggesting better recovery when IVIG treatment is initiated earlier [79,168]. However, once WNV encephalitis and TBE have entered the CNS, they may be more challenging to treat effectively with IVIG [175]. In a study by Piantadosi et al., IVIG was administered to a patient with Powassan virus (POWV) encephalitis, resulting in an excellent outcome despite severe MRI changes [177]. Another patient with POWV encephalitis and co-infection with Borrelia burgdorferi experienced severe meningoencephalitis and respiratory failure. The patient received supportive treatment with ceftriaxone for neuroborreliosis and empirically administered IVIG, which led to significant neurological recovery [178].

In the absence of specific antiviral treatment for severe arboviral neuroinfections, as well as supportive care, the decision to administer IVIG as a therapeutic option should be made on a case-by-case basis, taking into account current scientific and professional knowledge to assess the risks and benefits. Future research is necessary to establish well-defined indications for and the optimal dosing and timing of IVIG administration, as well as the potential benefits of IVIG in immunocompromised patients.

### 6.2. Prevention

Prevention of TBEV infection involves implementing general measures, including the pasteurization of milk, reducing the tick population, and adopting personal protection measures (using repellents; wearing appropriate clothing, such as long sleeves, long pants, and closed-toe shoes, to minimize exposed skin and make it more difficult for ticks to attach; and careful inspection and prompt removal of ticks, such as conducting thorough tick checks on the body after spending time in nature, particularly in tick-infested areas). These measures are aimed at minimizing the risk of exposure and transmission. Ticks should be removed promptly and properly using fine-tipped tweezers [3,136,149]. 

Active immunization is considered an optimal and highly effective method of preventing TBE for individuals who are exposed to endemic areas either professionally or recreationally. The immunization schedule typically involves a series of three doses for complete primary immunization administered at specific intervals (0, 1–3, and 9–12 months). Following the primary immunization, a booster dose is recommended after 3 years. Subsequent booster doses are administered every 3–5 years, with the specific interval determined based on the patient’s age [136,158,179,180]. Accelerated immunization schedules enable the rapid induction of an immune response and the establishment of a stable antibody titer [158,161,179]. 

The vaccine for TBE is recognized for its excellent tolerability, safety, and high effectiveness, ranging from 96% to 98.7%. Over the course of four decades, Austria has administered the TBE vaccine to over 35 million individuals, resulting in a substantial decline in TBE incidence and nearly eliminating the disease caused by TBEV [2,3,156,158,181]. 

The occurrence of the disease in vaccinated individuals has been reported by several authors, particularly among older individuals with a weakened immune response to the vaccine [180,181,182].

## 7. Diagnosis of Tick-Borne Encephalitis Virus Infection

Prompt and accurate diagnosis of the infection with the TBEV is crucial for appropriate management, prevention of complications, and adequate implementation of control measures. However, diagnosing TBE can be laden with challenges due to non-specific results in the initial disease stages, where abnormal liver function tests, decreased white blood cell counts, or reduced platelet counts may be observed [183]. Within a short time-frame following TBEV infection and the emergence of symptoms, a definitive diagnosis is habitually established by detecting IgM and IgG antibodies in the patient’s cerebrospinal fluid (CSF) and/or blood serum [184]. This is a standard approach and it has to be emphasized that the timing of antibody detection can vary depending on the individual’s immune response [3].

Consequently, some specific diagnostic criteria that primarily focus on serological testing for TBE have been put forth by the European Centre for Disease Control (ECDC). As per their guidelines, two criteria should be met for disease confirmation: the presence of clinical symptoms indicating inflammation of the central nervous system (CNS) and positive laboratory findings [185]. The latter can encompass several factors: the detection of TBE-specific IgM and IgG antibodies in blood serum; the identification of TBE-specific IgM or both IgM and IgG antibodies in the CSF, seroconversion or a notable rise in TBE-specific antibody levels observed in paired serum samples; the identification of viral genetic material in the blood, CSF, or other bodily fluids or tissues; and viral isolation [185]. A similar stance is taken by the European Academy of Neurology (EAN), which adds that the imaging of the brain/spinal cord has a valuable role in establishing a diagnosis of TBE, despite its limited sensitivity and specificity [4].

There are some limitations to serological testing; most notably, the potential cross-reactivity of antibodies, which should be considered in the interpretation phase as it can be a pertinent issue for the constituents of the genus *Flaviviridae* [186]. Erroneous interpretation of another CNS disease or infection may occur within several months of acute TBEV infection or following the initial primary immunization with two vaccine doses, since specific TBE IgM antibodies can still be detectable during this period [3,187]. Caution is also required when interpreting TBE serology results in patients who have been vaccinated against TBE and present with meningoencephalitis or meningitis. More specifically, in instances of vaccination breakthroughs, the serological response may differ from that observed in unvaccinated individuals with TBE, potentially leading to these cases being overlooked [3]. Vaccination breakthrough cases are typically defined as deferred development of IgM antibody response, as specific antibodies are usually not detectable during the inaugural week of the TBE meningoencephalitic phase; instead, there is a rapid increase in specific serum IgG antibodies [188,189].

Molecular techniques, such as reverse transcription–polymerase chain reaction (RT-PCR), can be employed to detect TBEV RNA in patient samples. Notably, viral presence can only be ascertained during the acute disease phase; however, affected individuals are typically admitted to the hospital when neurological symptoms manifest, which is usually beyond the point when TBE viral nucleic acid is detectable in the blood or CSF [190]. This means the diagnostic yield of molecular methods is limited, but in areas where multiple tick-borne diseases are prevalent, the inclusion of TBE RT-PCR testing in the diagnostic algorithm for febrile disease after a tick bite could prove to be a valuable diagnostic approach [3,191]. Albeit rare, food-borne transmission of TBEV is a possibility, which prompted a recent validation of the RT-qPCR method for detecting this virus in raw milk products [93,192]. It also has to be emphasized that collecting and screening ticks using real-time RT-PCR cannot be endorsed for human TBE risk assessment [193].

Several biomarkers can be used to support the diagnosis of TBE in the clinical milieu. In accordance with the current understanding, CSF testing/analysis plays a pivotal role, as it can reveal moderate pleocytosis characterized by an increased segmented granulocyte count, as well as elevated protein concentration [187]. As the disease progresses, there is a shift towards the predominance of lymphocytes. Such elevated lymphocyte counts can persist for several weeks, even after clinical improvement has occurred [194]. However, some authors caution that testing for TBEV infection should be considered in individuals presenting with encephalitis symptoms consistent with TBE even in the absence of CSF pleocytosis, particularly if there is a known risk of tick exposure in a region endemic for TBE [195].

Furthermore, C-reactive protein or procalcitonin levels are measured to aid in differentiating TBEV infection from other neuroinflammatory disorders of bacterial origin. More specifically, the levels of these proteins are generally lower in TBE compared to bacterial CNS infections [2,3,136]. Still, it is important to note that these additional biomarkers only provide supportive evidence and do not independently confirm the diagnosis of TBE. A recent review pinpointed several candidate biomarkers that can be used in the TBE diagnostic, prognostic, and monitoring stages; these include chemokines such as CCL2, CCL7, CXCL1, CXCL2, CXCL9, and CXCL12 [196].

In fact, cytokines and chemokines have been investigated as potential diagnostic biomarkers for the assessment of the severity of TBE, such as in predicting the presence of encephalitis (either moderate or severe) compared to primarily meningeal symptoms and differentiating TBE meningoencephalitis from meningitis in both adults and children [197,198,199]. Such findings indicate that they could be used to analyze and forecast the disease course. A recent study from Croatia demonstrated that patients with this disease more frequently present with higher levels of IFN-gamma and IL-6 in the CSF compared to controls, while IL-6 can also be detected in urine specimens in much higher concentrations than in serum samples [199]. Due to the heterogeneity of the literature data thus far, an optimal diagnostic set of biomarkers has yet to be determined for TBEV infection.

A recent cross-sectional study in Germany by Schley et al. [200] suggested that individuals displaying typical symptoms of TBE may be under-tested, which consequently results in the potential under-diagnosis of TBE cases. In addition, a lack of standardized TBE testing protocols in hospitals (and even in departments of the same institution) has been identified as an issue that may be relevant for many countries [200]. In order to guarantee accurate identification of cases, these authors recommend that TBE testing become standardized and a routine practice consistently employed for all patients exhibiting relevant symptoms or who have been exposed to common risk factors associated with TBE.

Here, it has to be emphasized that tick vectors or vertebrate reservoir hosts show a propensity to carry multiple microorganisms that can cause illness in humans (e.g., *Babesia*, *Anaplasma*, and *Borrelia* species alongside viral agents such as TBEV); conversely, human co-infections can be interpreted in various manners, spanning from individuals testing positive for antibodies without experiencing clinical symptoms to the simultaneous occurrence of clinical manifestations stemming from infections by two tick-borne microorganisms [201]. Naturally, this comes with implications for diagnostic approaches, as certain bacterial infections can present with clinical manifestations and CSF findings similar to viral infections such as TBEV. A recent systematic review has shown that the majority of co-infection cases involved patients who had one tick-borne disease while also having antibodies against another tick-borne microorganism [202]. Furthermore, co-disease was particularly common in two scenarios: among individuals with high fever and erythema migrans as clinical symptoms and patients experiencing neurological symptoms associated with TBEV or neuroborreliosis [202]. The most frequent putative association was demonstrated between TBEV and *Borrelia burgdorferi sensu lato*; thus, caution is necessary when interpreting serological results (particularly in instances of overlapping symptoms) [202].

## 8. Conclusions and Perspectives

The expanding risk areas, increasing incidence of TBEV infections, and emerging endemic regions emphasize the importance of gaining a better understanding of the factors influencing TBEV pathogenesis. In this comprehensive review, we have provided insights into the virus’s structure, reproduction, transmission, pathogenesis, epidemiology, risk factors, clinical manifestations, and diagnostic approaches. However, further research is crucial to deepen our knowledge.

Notably, the neurovirulence and neuroinvasiveness of TBEV can be significantly impacted by mutations in its infectious genomic RNA, underscoring the need for genome-focused investigations [40,44,45,46,47]. The geographic distribution of TBEV is predominantly determined by the distribution of its tick vectors. While *Ixodes ricinus* and *Ixodes persulcatus* ticks are the main vectors for TBEV-Eu, TBEV-Sib, and TBEV-FE, recent studies have identified at least 22 other tick species capable of carrying and transmitting the virus [72,78,79,80,81,82. Furthermore, birds play a role in spreading infected ticks to new areas, expanding the potential for transmission [72,89].

Several factors contribute to the increasing occurrence and spread of TBEV, both within and outside endemic areas [132,133]. Climate change, such as global warming, impacts TBEV reservoir and vector populations, affecting tick biology and ecology [134]. Mild winters and earlier springs can boost tick populations and their development [78,127,137]. Additionally, travel patterns and consumption of unpasteurized milk and milk products play roles in TBEV transmission [93,121].

With the incidence of TBEV reaching 0.9 cases per 100,000 people in 2020, understanding these aspects is crucial. The highest incidence rates in recent years have been observed in Lithuania, Slovenia, the Czech Republic, and Latvia [120]. It is imperative to recognize and acknowledge that accurate and timely diagnosis of TBEV infection is vital due to the non-specific nature of the initial symptoms [164]. Despite the challenges it presents, accurate diagnosis is essential for effective management, prevention of complications, and the implementation of control measures [181]. By deepening our knowledge of TBEV and its associated factors, we strive to enhance strategies for timely diagnosis, appropriate management, and effective control measures against TBEV infections.

## Figures and Tables

**Table 1:** Putative pathways by which TBEV penetrates the blood–brain barrier.

No.	Pathway	Ref.
1.	Via neurons after infection of the peripheral nerves	[108]
2.	Infection of olfactory neurons	[112]
3.	Infection of the capillary endothelium and transcytosis into the brain parenchyma	[111]
4.	By diffusion from capillary endothelial cells through the highly permeable blood–brain barrier	[108]

## Data Availability

The authors can provide the data if needed.
